# Heavy Hitters, Light Sleepers: Collision Frequency and Locomotor Load on Sleep Architecture in Professional Rugby Union Players

**DOI:** 10.1002/ejsc.70052

**Published:** 2025-08-30

**Authors:** Kanon Uchiyama, Peter Peeling, Shona L. Halson, Machar Reid, Karen Wallman, Jennifer Walsh, Simon Thomas, Olivier Girard

**Affiliations:** ^1^ School of Human Sciences (Exercise and Sport Science) The University of Western Australia Perth Australia; ^2^ Western Force Perth Australia; ^3^ School of Behavioural and Health Sciences McAuley at Banyo Australian Catholic University Brisbane Australia; ^4^ Centre for Sleep Science School of Human Sciences The University of Western Australia Crawley Australia; ^5^ Department of Pulmonary Physiology & Sleep Medicine West Australian Sleep Disorders Research Institute Sir Charles Gairdner Hospital Nedlands Australia

**Keywords:** collisions, match demands, rugby, sleep architecture, team‐sport athletes

## Abstract

To assess whether certain players are more vulnerable to postmatch sleep disturbances by examining the relationship between match demands—collision frequency and locomotor load—and sleep in professional male rugby union players. A linear mixed‐effects regression examined the relationship between match variables and sleep in 13 rugby players across three matches. Match variables included six physical demand variables derived from video analysis and GPS data (collision frequency, total distance, high‐speed distance, sprint distance, acceleration load and fast acceleration count) and two contextual variables (location and kick‐off time). Sleep variables collected via home‐based polysomnography included total sleep time, sleep efficiency, sleep onset/offset, sleep onset latency, wake after sleep onset, number of awakenings and sleep stages (light, deep and rapid eye movement sleep [REM], evaluated both by proportion [%] and time [min]). Each match collision decreased total sleep time (*β* = −4 ± 1 min and *p =* 0.006) and REM sleep (time: *β* = −2 ± 0 min and *p <* 0.001; proportion: *β* = −0.6 ± 0.2% and *p* = 0.021). Conversely, every 500 m increase in locomotor load (total distance) increased REM sleep (time: *β* = +6 ± 2 min and *p =* 0.014; proportion: *β* = +2.7 ± 0.6% and *p* = 0.002). Every 100 m increase in high‐speed distance was associated with decreased REM sleep time (*β* = −7 ± 3 min and *p =* 0.020). Match demands, including collision frequency and locomotor load, were associated with changes in postmatch sleep architecture in professional rugby players, particularly REM sleep. Furthermore, greater number of collisions was associated with reduced sleep quantity. Practitioners can leverage GPS and video analysis data to tailor additional sleep strategies aimed at improving postmatch sleep based on individual match demands.

## Highlights

- Players exposed to greater collisional demands may require additional evidence‐based sleep strategies to compensate for reductions in total sleep duration and rapid eye movement sleep.
- Beyond collisions, locomotor demands need to be considered. Greater total distance may support rapid eye movement sleep, whereas excessive high‐speed running (> 5 m.s^−1^) could compromise it.
- Training and recovery schedules could be tailored using GPS data and video analysis to identify players exposed to elevated collisional activity and/or high‐intensity locomotor demands.
- Sleep interventions should be individualised within teams, considering both physical match demands and contextual factors, including match schedule and location.

## 1 Introduction

Assessing sleep architecture, including sleep stages, offers deeper insight into sleep quality, given the distinct roles these stages play in athletes' next‐day function. For example, deep sleep drives the release of ∼95% of daily growth hormone (Czeisler and Klerman 1999), supporting an athlete's ‘physical recovery’ through muscle repair, bone remodelling (Rooyackers and Nair 1997) and immune system strengthening (Besedovsky et al. 2012). Meanwhile, REM sleep, marked by heightened neural activity, underpins learning, memory consolidation and emotional regulation (K. E. Miller and Gehrman 2019), whilst also reducing norepinephrine levels—a chemical linked to stress and emotions (Pilcher and Huffcutt 1996)—facilitating an athlete's ‘mental recovery’.

Field‐based sleep research on professional team‐sport athletes shows that sleep patterns often become irregular during the competitive season (Fullagar et al. 2015). Specifically, after a match, both sleep quantity and quality (an individual's self‐satisfaction with aspects of sleep; sleep duration, sleep onset latency, wake after sleep onset and sleep efficiency) are disrupted (Dunican et al. 2018; Sim et al. 2023). Previous studies have primarily relied on validated wrist activity monitors for dichotomous sleep‐wake analyses (Halson 2019; Driller et al. 2023). Compared to nights after training or rest, players often experience delayed sleep onset, reduced sleep duration and greater sleep fragmentation after a match, along with frequent self‐reports of sleep dissatisfaction the following morning (Dunican et al. 2018; Sim et al. 2023; Shearer et al. 2015). However, less is known about how competitive matches impact sleep architecture, including the different stages of sleep (i.e., light, deep and rapid eye movement [REM] sleep).

Although wrist activity monitors provide ecological validity in field research, they cannot measure sleep architecture, which laboratory polysomnography (PSG)—the gold standard for sleep assessment—can identify. This limitation has hindered our understanding of athletes' sleep in field settings. Nonetheless, recent innovations in wireless portable home‐based PSG devices and commercial technology now enable the measurement of sleep architecture in the field (Halson 2019; Driller et al. 2023; D. J. Miller et al. 2022). Considering the differing roles of sleep stages for recovery, field‐based researchers are adopting these new approaches to explore athletes' sleep architecture (Driller et al. 2023; Aloulou et al. 2020; Vitale et al. 2022) uncovering potential priorities for recovery.

To date, three field studies have examined postmatch sleep architecture in team‐sport athletes. Two of these utilised home‐based PSG devices (D. J. Miller et al. 2022; Aloulou et al. 2020), whereas one employed a commercial sleep device with surrogate plethysmography to infer sleep architecture (Sanders et al. 2024). Findings have been mixed, potentially due to variations in sample characteristics, including playing level and sport, as well as the sleep device used. For instance, two studies reported significant disruptions to sleep patterns after a match in soccer (Sanders et al. 2024), volleyball, basketball and field hockey athletes (Aloulou et al. 2020), across mixed playing levels (Tier 3 to 4 athletes as classified by McKay et al. (2022)), yet both found no changes in sleep architecture. Contrastingly, the third study, investigating youth rugby union players, identified alterations in sleep architecture related to match demands (D. J. Miller et al. 2022). Specifically, players who covered greater high‐speed running distances and had fewer collisions exhibited greater deep sleep and reduced light sleep (D. J. Miller et al. 2022). These findings suggest that match demands, potentially influenced by player position, may lead to varying changes in sleep characteristics the night after a match.

Postmatch sleep disruptions are typically attributed to various competition‐related factors, including fixture schedules, especially evening matches that interfere with the natural downregulation needed to initiate sleep (due to stadium floodlights and heightened arousal), and travel for away matches. Athlete behaviours both before (e.g., caffeine ingestion) and after matches (e.g., social, press and fan engagements) also contribute to these disruptions (Dunican et al. 2018; Shearer et al. 2015; Nédélec et al. 2019). These factors can reduce sleep opportunities and ultimately disrupt recovery (Dunican et al. 2018; Sim et al. 2023). However, although postmatch sleep disturbances are commonly experienced by teams, there appears to be high variability in sleep responses amongst players within a team, and therefore, a need for individualised approaches (Dunican et al. 2018; Vitale et al. 2022; Uchiyama et al. 2025).

Rugby union is an 80‐min high‐intensity, intermittent collision sport, involving repeated bouts of high‐intensity efforts, such as running, tackling, rucking, mauling and scrummaging, interspersed with lower‐intensity recovery periods (Roberts et al. 2008). In rugby union, practitioners often assess match demands using a two‐dimensional approach, examining both collisions (i.e., contact load) and locomotor performance (i.e., run load), as these vary significantly by position (Roberts et al. 2008; Lindsay et al. 2015). Although the latter provides us with some insight into potential stimuli for exercise‐induced muscle damage, the impact‐induced muscle damage derived from contact sports is also an important consideration (Naughton et al. 2018). Forwards typically have greater contact load and lower locomotor load, whereas backs engage in more running but less contact (Roberts et al. 2008; Lindsay et al. 2015). To fully understand the physical demands placed on rugby players and their effects on sleep, it is crucial to consider both contact and locomotor loads. Although current technology has limitations to quantify collision intensity, the frequency of collisions can be quantified via video analysis (Roberts et al. 2008), typically alongside global positioning systems (GPS)‐derived locomotor load to assess physical demands.

Beyond the aforementioned research (Aloulou et al. 2020), only one other study has assessed the relationship between match demands (i.e., collision frequency and locomotor load) and subsequent sleep, using dichotomous sleep‐wake analysis (Leduc et al. 2022). This study found that, alongside factors, such as kick‐off times and travel, more collisions during matches resulted in players waking later the next morning (Leduc et al. 2022). Although both studies (Aloulou et al. 2020; Leduc et al. 2022) recruited Tier 3 athletes (McKay et al. 2022), there appear to be significant associations between match demands and the subsequent sleep of rugby union players. Given that match demands are typically greater at the professional‐level (Tier 4 and 5 athletes) (McKay et al. 2022; Sirotic et al. 2009), along with increased travel requirements and later kick‐off times due to broadcasting schedules (Fullagar et al. 2015), professional rugby teams are likely to face even greater sleep disruptions. Hence, there is a pressing need to investigate this in professional cohorts.

We aimed to examine the relationship between match demands and postmatch sleep of professional male rugby union players. From the existing literature (Aloulou et al. 2020; Leduc et al. 2022), we hypothesised that match demands would be associated with alterations in sleep architecture, with: (1) more collisions increasing light sleep, and consequently reducing deep and REM sleep; (2) greater run load, especially at higher intensity, promoting deep sleep, thereby reducing light and REM sleep and (3) contextual factors, such as away matches and later kick‐off times, delaying sleep onset and offset times.

## 2 Methods

### 2.1 Study Design

This retrospective study examines the relationship between physical match demands and subsequent sleep in professional rugby union players. Sleep data from male professional rugby union players contracted for the 2023 Super Rugby season, collected exclusively on postmatch nights, were analysed alongside eight match variables representing either physical demands or contextual factors. Analysis was limited to players who were named in the matchday line‐up. Players participated in four field sessions (totalling 6 h of rugby practice) and three to four gym sessions (4.5–4.75 h) weekly. Informed consent was obtained, and ethics approval was granted by the host institution (2022/ET000700).

### 2.2 Sleep

Sleep data were collected using a wireless home‐based PSG device (Somfit, Compumedics, Melbourne, Australia), which players wore on their forehead during designated sleep assessment nights. The Somfit is validated against PSG with an agreement of 63%–79% (D. J. Miller et al. 2022; Roach et al. 2025) and has shown to measure sleep with better accuracy than alternate commercial sleep wearables (i.e., wrist‐ or finger‐worn trackers) that are commonly utilised by athletes due to its forehead placement (i.e., electroencephalography signals) (Shearer et al. 2015). Players were instructed to wear the device immediately before bed and remove it upon waking (once out of bed). Data were uploaded to the proprietary viewing platform (Nexus360; Compumedics, Melbourne, Australia) and automatically analysed. Sleep staging was then manually scored in 30‐s epochs as N1, N2, N3 and REM sleep (or wake) by a single experienced sleep scientist according to the recommended AASM criteria (Troester et al. 2023). All recordings were deemed of good‐capture quality (> 80% epochs) (Roach et al. 2025), with scorable electroencephalography data available for 99 ± 3% of epochs. All sleep parameters were defined with manual scoring criteria, which are included in Table S1.

### 2.3 Match Demand: Collision Frequency and Locomotor Load

Six of the eight match variables were related to physical demands. Collision frequency was one of the six variables, summed from coded collision events (frequency count of carries, ruck involvements and offensive and defensive tackles), similar to previous studies (Aloulou et al. 2020; Leduc et al. 2022). Collision events were obtained from match‐play records provided by Opta (Stats Perform, London, United Kingdom), which uses human annotation to track player actions during matches (Aloulou et al. 2020; Leduc et al. 2022). Opta has shown strong interoperator reliability (kappa value = 0.94) (Liu et al. 2013) and rugby analysts are recruited only after achieving 95% accuracy against the Opta analysis system (Parmar 2017).

The remaining five physical match demand variables relate to locomotor load and were obtained from validated global positioning system (GPS) units (10 Hz Vector S7, Catapult Sports, Melbourne, Australia) worn by players during all matches (Crang et al. 2022, 2024). The device has shown good reliability for distance‐, velocity‐banded distance and acceleration‐related metrics (CV of 0.1%–3.9%) (Crang et al. 2022). Metrics included total distance, high‐speed distance (> 5 m.s^−1^), sprint distance (> 7 m.s^−1^), acceleration load and the count of fast accelerations (> 2.5 m.s^−2^) each calculated as separate variables. These metrics were selected as they are commonly used by practitioners to profile match‐play demands in team‐sports (Sheehan et al. 2022; Dawson et al. 2024).

### 2.4 Match‐Contextual Factors

Two additional variables were related to the context of the match (location and time). Matches were coded as either home or away based on location, alongside scheduled kick‐off time (hh:mm). The three matches took place at the following local kick‐off times, locations and time zones: 19:00 (home; GMT+8), 15:00 (away; GMT+11) and 15:35 (away; GMT+13), corresponding to rounds one, two and six of the competition.

### 2.5 Statistical Analysis

**FIGURE 1:**
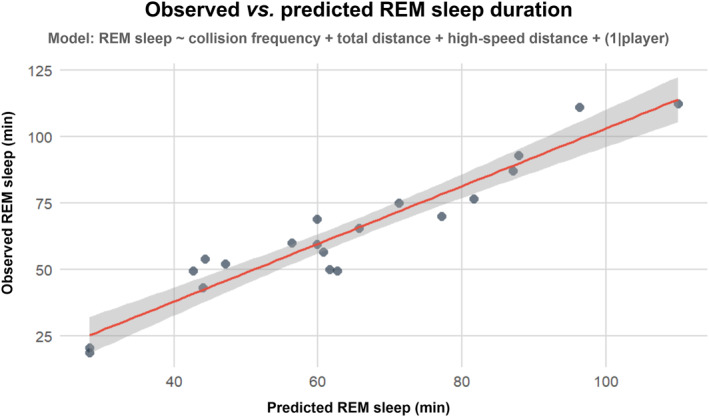
Observed versus predicted REM sleep duration from a multivariate model with significant predictors (collision frequency, total distance and high‐speed distance) as covariates and *player* as a random intercept. The red line denotes the predicted REM sleep duration, whereas the dots show the observed individual data points.

All statistical analyses were completed with R (Version 4.0). Initially, Pearson's *r* correlational analysis assessed multicollinearity among physical demand match variables leading to the removal of strongly correlated (*r >* 0.85) variables (i.e., acceleration load and fast accelerations; Table 1) to ensure a robust linear regression model (Christ 2009). A linear mixed‐effects regression analysis was then conducted to examine the associations between remaining match variables and sleep. This model incorporated fixed covariates effects as match variables (match demand and match‐contextual variables) while accounting for individual differences (*player* as a random intercept) on RStudio using the (lmerTest) package. Results are presented as estimate coefficients (*β*) ± standard error of the estimate (SE), including 95% confidence intervals of the estimate (CI), as well as the effect sizes (*η*_*p*_^2^) and *p‐*values of each match variable. Statistical significance was set at *p* < 0.05. Effect sizes were interpreted as *small* effect ≤ 0.01, *moderate* effect = 0.06 and *large* effect ≥ 0.14 (Cohen 1977). A separate ANOVA analysis, using the same package, was conducted to assess the differences in match demand variables between positional groups (fixed covariate; forwards vs. backs), accounting for individual differences by including *player* as a random intercept. The strongest predictive model, identified based on model fit and predictor significance, was further examined using a post hoc multivariate analysis to assess the relationship between observed and predicted values (Figure 1).

**TABLE 1:** Correlational analysis of physical demand match variables to assess multicollinearity (*r*).

	Contact load, *n*	Total distance, m	High‐speed running, m	Sprint distance, m	Acceleration load, AU	Fast accelerations, *n*
Contact load, *n*	—	0.493^a^	0.013	−0.034	0.486^a^	0.148
Total distance, m	0.493^a^	—	0.712^a^	0.450^a^	0.980^a^ ^,^ ^b^	0.669^a^
High‐speed distance, m	0.013	0.712^a^	—	0.581^a^	0.755^a^	0.874^a^ ^,^ ^b^
Sprint distance, m	−0.034	0.450^a^	0.581^a^	—	0.384	0.398
Acceleration load, AU	0.486^a^	0.980^a^ ^,^ ^b^	0.755^a^	0.384	—	0.744^a^
Fast accelerations, *n*	0.148	0.669^a^	0.874^a^ ^,^ ^b^	0.398	0.744^a^	—

^a^ Significant correlation between match demand predictor variables (*p* < 0.05).

^b^ Strong correlation between match demand predictor variables (*r* > 0.85).

## 3 Results

Sleep data from 13 players were analysed, including seven forwards (age: 27.4 ± 4.8 years; height: 190.6 ± 6.2 cm and body weight: 111.0 ± 7.3 kg) and six backs (age: 28.7 ± 2.9 years; height: 180.8 ± 3.9 cm and body weight: 89.3 ± 6.4 kg). A total of 20 nights of postmatch sleep data, collected across three matches, were included in the analysis. This comprised seven single‐match observations, five players with data from two matches and one player with data from all three matches.

### 3.1 Match Demands and Sleep of Professional Male Rugby Union Players

Match demands and subsequent sleep values of players were categorised into positional groups: forwards versus backs in Table 2. The average time on field players spent during match play was 48 + 23 min. On average, players fell asleep at 01:02 ± 01:17 a.m. (forwards: 01:22 ± 00:59 a.m. and backs: 00:38 ± 01:32 a.m.) and woke at 06:01 ± 01:07 a.m. (forwards: 06:29 ± 00:55 a.m. and backs: 06:46 ± 01:14 a.m.).

**TABLE 2:** Match demands (top), sleep characteristics (middle) and sleep architecture (bottom) of professional male rugby union players (*n* = 13) from three matches, categorised into positional groups (forwards vs. backs).

Match demands						
	Contact load, *n*	Total distance, m	High‐speed distance, m	Sprint distance, m	Acceleration load, AU	Fast accelerations, *n*
Overall	17 ± 11	5056 ± 1701	386 ± 250	49 ± 68	1592 ± 519	33 ± 15
	(4–39)	(2019–8394)	(47–818)	(0–229)	(732–2621)	(8–60)
Forwards	23 ± 12^a^	4707 ± 1755	264 ± 225	25 ± 58	1481 ± 544	27 ± 15
	(4–39)	(2019–7477)	(47–784)	(0–193)	(732–2399)	(8–57)
Backs	10 ± 4	5482 ± 1627	536 ± 199^b^	79 ± 70	1727 ± 484	40 ± 13
	(6–19)	(3737–8394)	(223–818)	(0–229)	(1031–2621)	(14–60)

Sleep characteristics						
Sleep	Sleep duration, min	Sleep efficiency, %	Sleep onset latency, min	Wake after sleep onset, min	Awakenings, *n*	REM latency, min
Overall	317 ± 54	87 ± 8	31 ± 30	27 ± 16	18 ± 7	123 ± 42
	(181–388)	(60–96)	(1–105)	(7–72)	(8–31)	(66–187)
Forwards	288 ± 53	86 ± 9	36 ± 31	24 ± 11	17 ± 7	141 ± 33
	(181–367)	(60–96)	(5–105)	(7–40)	(8–31)	(84–187)
Backs	352 ± 29	88 ± 7	25 ± 29	31 ± 21	20 ± 6	100 ± 42
	(303–388)	(71–95)	(1–84)	(11–72)	(12–29)	(66–176)

Sleep architecture						
	Light sleep, %	Light sleep, min	Deep sleep, %	Deep sleep, min	REM sleep, %	REM sleep, min
Overall	61 ± 7	193 ± 35	19 ± 5	61 ± 20	20 ± 6	64 ± 25
	(51–75)	(127–245)	(10–27)	(33–103)	(9–37)	(19–113)
Forwards	65 ± 7	186 ± 41	19 ± 5	53 ± 13	17 ± 4	50 ± 17
	(54–75)	(127–245)	(10–27)	(33–82)	(9–23)	(19–69)
Backs	57 ± 5	201 ± 27	20 ± 6	71 ± 23	23 ± 7	81 ± 23
	(51–65)	(156–233)	(10–27)	(37–103)	(13–37)	(50–113)

*Note:* Data are presented as mean ± sd with the range below (minimum–maximum).

Abbreviation: AU, arbitrary units.

^a^ Significant difference between positional groups (*p* < 0.05), whereby forwards present larger values than backs.

^b^ Significant difference between positional groups (*p* < 0.05), whereby backs present larger values than forwards.

### 3.2 Effect of Match Demands on Sleep

Match demand variables for both collision frequency and locomotor load and their associations with sleep variables are presented in Table 3.

**TABLE 3:** Results of the linear mixed‐effects model analysis examining the associations between match variables and sleep variables in professional male rugby union players (*n* = 13) across three matches.

Sleep variable	Fixed effects	*β*	SE	95% CI		ES (*η*_*p*_^2^)	*p*
Sleep duration	(Intercept)	412.12	99.17	217.76	606.48	—	**0.001**
	Contact load	−3.46	1.07	−5.56	−1.37	0.45	**0.006**
	Total distance	−0.01	0.01	−0.02	0.01	0.02	0.604
	High‐speed distance	0.05	0.06	−0.07	0.17	0.05	0.423
	Sprint distance	0.04	0.17	−0.30	0.37	0.00	0.825
	Location (home)	−2.35	32.24	−65.54	60.85	0.00	0.943
	Kick‐off	−41.16	154.06	−343.12	260.80	0.01	0.794
Sleep efficiency, %	(Intercept)	124.95	21.01	83.78	166.13	—	**<** **0.001**
	Contact load	−0.04	0.26	−0.54	0.47	0.00	0.887
	Total distance	0.00	0.00	−0.01	0.00	0.08	0.341
	High‐speed distance	0.01	0.01	−0.02	0.04	0.07	0.522
	Sprint distance	0.01	0.04	−0.07	0.08	0.00	0.854
	Location (home)	14.07	6.86	0.62	27.52	0.27	0.064
	Kick‐off	−50.04	32.08	−112.92	12.84	0.21	0.153
Light sleep, min	(Intercept)	331.90	79.83	175.48	488.40	—	**0.001**
	Contact load	−0.94	0.86	−2.63	0.75	0.08	0.296
	Total distance	−0.01	0.01	−0.03	0.00	0.16	0.146
	High‐speed distance	0.07	0.05	−0.03	0.16	0.13	0.190
	Sprint distance	−0.10	0.14	−0.37	0.17	0.04	0.476
	Location (home)	14.96	25.96	−35.92	65.83	0.02	0.574
	Kick‐off	−122.40	124.00	−365.50	120.65	0.07	0.342
Deep sleep, min	(Intercept)	54.70	48.44	−40.24	149.63	—	0.307
	Contact load	−0.23	0.61	−1.43	0.96	0.07	0.743
	Total distance	−0.01	0.01	−0.02	0.00	0.14	0.301
	High‐speed distance	0.05	0.03	−0.02	0.12	0.54	0.297
	Sprint distance	0.01	0.09	−0.17	0.19	0.00	0.919
	Location (home)	−13.17	15.85	−44.25	17.90	0.09	0.433
	Kick‐off	36.74	73.71	−107.73	181.22	0.07	0.649
REM sleep, min	(Intercept)	38.60	42.36	−44.43	121.62	—	0.379
	Contact load	−2.12	0.46	−3.02	−1.23	0.62	**<** **0.001**
	Total distance	0.01	0.00	0.00	0.02	0.38	**0.014**
	High‐speed distance	−0.07	0.03	−0.12	−0.02	0.35	**0.020**
	Sprint distance	0.15	0.07	0.00	0.29	0.24	0.067
	Location (home)	−1.85	13.77	−28.84	25.15	0.00	0.895
	Kick‐off	28.26	65.81	−100.73	157.25	0.01	0.675
Light sleep, %	(Intercept)	75.04	9.10	57.19	92.88	—	**<** **0.001**
	Contact load	0.22	0.19	−0.15	0.60	0.10	0.269
	Total distance	0.00	0.00	−0.01	0.00	0.20	0.094
	High‐speed distance	0.01	0.01	−0.01	0.03	0.10	0.306
	Sprint distance	−0.02	0.02	−0.07	0.02	0.09	0.296
	Location (home)	5.01	2.98	−0.83	10.85	0.36	0.152
	Kick‐off	−15.19	12.20	−39.09	8.71	0.29	0.285
Deep sleep, %	(Intercept)	13.28	11.83	−9.90	36.47	—	0.315
	Contact load	0.30	0.21	−0.11	0.71	0.20	0.184
	Total distance	−0.002701	0.00	−0.01	0.00	0.19	0.123
	High‐speed distance	0.02	0.01	−0.01	0.04	0.20	0.256
	Sprint distance	0.01	0.03	−0.04	0.06	0.01	0.689
	Location (home)	−4.66	3.95	−12.40	3.08	0.23	0.295
	Kick‐off	14.38	16.93	−18.80	47.56	0.20	0.460
REM sleep, %	(Intercept)	8.62	9.98	−10.93	28.17	—	0.448
	Contact load	−0.57	0.16	−0.88	−0.25	0.75	**0.021**
	Total distance	0.01	0.00	0.00	0.01	0.64	**0.002**
	High‐speed distance	−0.03	0.01	−0.04	−0.01	0.72	0.057
	Sprint distance	0.02	0.02	−0.02	0.06	0.06	0.379
	Location (home)	0.02	3.33	−6.49	6.54	0.00	0.995
	Kick‐off	5.07	14.60	−23.54	33.69	0.06	0.763
Sleep onset, hh:mm	(Intercept)	0.97	0.11	0.76	1.18	—	**<** **0.001**
	Contact load	0.00	0.00	0.00	0.00	0.07	0.335
	Total distance	0.00	0.00	0.00	0.00	0.11	0.230
	High‐speed distance	0.00	0.00	0.00	0.00	0.07	0.356
	Sprint distance	0.00	0.00	0.00	0.00	0.01	0.753
	Location (home)	0.08	0.03	0.01	0.15	0.29	**0.037**
	Kick‐off	−0.04	0.17	−0.36	0.29	0.00	0.829
Sleep offset, hh:mm	(Intercept)	0.28	0.08	0.12	0.44	—	**0.005**
	Contact load	0.00	0.00	0.00	0.00	0.20	0.254
	Total distance	0.00	0.00	0.00	0.00	0.10	0.300
	High‐speed distance	0.00	0.00	0.00	0.00	0.08	0.491
	Sprint distance	0.00	0.00	0.00	0.00	0.03	0.580
	Location (home)	0.08	0.03	0.03	0.13	0.44	**0.011**
	Kick‐off	−0.07	0.13	−0.32	0.17	0.03	0.577
Sleep onset latency, min	(Intercept)	−83.84	74.99	−230.81	63.14	—	0.284
	Contact load	0.40	0.81	−1.19	1.98	0.02	0.633
	Total distance	0.01	0.01	−0.01	0.02	0.09	0.278
	High‐speed distance	−0.05	0.05	−0.14	0.04	0.08	0.298
	Sprint distance	0.03	0.13	−0.22	0.28	0.00	0.830
	Location (home)	−40.27	24.38	−88.06	7.51	0.17	0.123
	Kick‐off	139.81	116.50	−88.53	368.14	0.10	0.252
Wake after sleep onset, min	(Intercept)	−1.19	37.55	−74.80	72.41	—	0.975
	Contact load	−0.87	0.58	−2.00	0.26	0.19	0.162
	Total distance	0.01	0.00	0.00	0.01	0.11	0.231
	High‐speed distance	−0.01	0.03	−0.08	0.05	0.02	0.667
	Sprint distance	−0.05	0.08	−0.21	0.10	0.03	0.512
	Location (home)	−19.39	12.48	−43.86	5.08	0.19	0.151
	Kick‐off	40.40	55.45	−68.29	149.08	0.07	0.488
Awakenings, *n*	(Intercept)	16.17	18.94	−20.95	53.28	—	0.409
	Contact load	−0.06	0.20	−0.46	0.34	0.01	0.773
	Total distance	0.00	0.00	−0.01	0.00	0.06	0.392
	High‐speed distance	0.00	0.01	−0.02	0.03	0.01	0.684
	Sprint distance	0.00	0.03	−0.06	0.06	0.00	0.993
	Location (home)	0.54	6.16	−11.53	12.61	0.00	0.931
	Kick‐off	13.92	29.42	−43.74	71.58	0.02	0.644
REM onset latency, min	(Intercept)	91.48	92.76	−90.33	273.29	—	0.367
	Contact load	2.00	1.12	−0.20	4.20	0.71	0.283
	Total distance	−0.02	0.01	−0.04	0.00	0.48	0.056
	High‐speed distance	0.12	0.06	0.00	0.25	0.75	0.265
	Sprint distance	−0.25	0.17	−0.58	0.09	0.29	0.206
	Location (home)	1.46	30.28	−57.88	60.81	0.00	0.963
	Kick‐off	107.07	141.91	−171.06	385.20	0.15	0.504

*Note:* The table presents the estimated coefficients (*β*), standard errors (SE), 95% confidence intervals (CI), effect sizes (*η*_*p*_^2^) and *p*‐values (bolded if *p* < 0.05).

**FIGURE 2:**
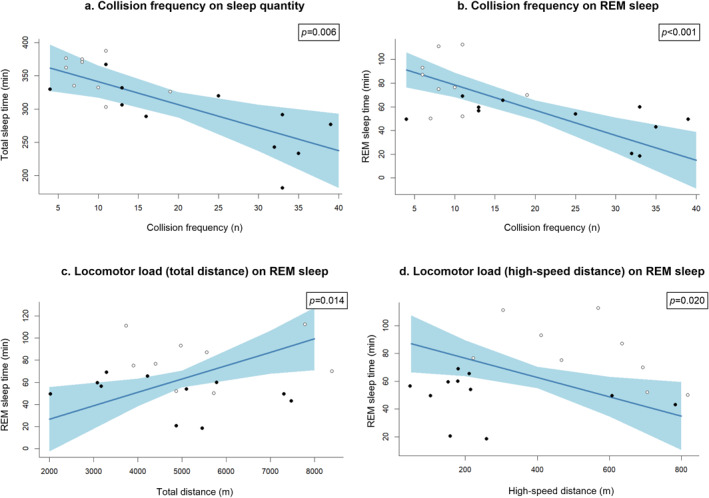
Significant associations (*p <* 0.05) between match demand variables and sleep variables in a professional male rugby union team (*n* = 13), including (a) collision frequency and total sleep time, (b) collision frequency and REM sleep time, (c) locomotor load (total distance) and REM sleep time and (d) high‐speed distance and REM sleep time. The blue line represents the estimated effect of the match variable on the sleep variable when all other variables are held constant. The direction of the line shows the nature of the relationship. The shaded area, in light blue, represents the confidence interval (95%) around the effect estimate. The dots represent the individual data points colour‐coded into positional groups (black: forwards and white: backs).

The model revealed significant associations between sleep variables and both collision frequency and locomotor load (Figure 2). For each player collision during a match, there were reductions in total sleep time (*β* = −4 ± 1 min and *p* = 0.006) as well as REM sleep time (*β* = −2 ± 0 min and *p* < 0.001) and proportion (*β* = −0.6 ± 0.2% and *p* = 0.021). Every additional 500 m of total distance covered increased REM sleep time (*β* = +6 ± 2 min and *p* = 0.014) and proportion (*β* = +2.7 ± 0.6% and *p* = 0.002). For every 100 m increase in high‐speed distance, REM sleep time decreased (*β* = −7 ± 3 min and *p* = 0.020).

### 3.3 Effect of Match Context on Sleep

The effects of match schedule (kick‐off times) and location (home or away) are presented in Table 3. Kick‐off times (19:00, 15:00 and 15:35 local time) did not significantly affect any sleep variables (*p >* 0.05). Playing at home was associated with later sleep onset (*β* = +1.9 ± 0.8 h and *p =* 0.037) and offset times (*β* = +1.9 ± 0.6 h and *p =* 0.011) compared to away matches. However, match location did not influence any other sleep variables.

## 4 Discussion

This study is the first to examine the relationship between physical match demands (collision frequency and locomotor load) and subsequent sleep architecture in professional male rugby union players. Our analysis of three in‐season matches indicates that the physical demands and match location were significantly associated with changes to postmatch sleep, particularly REM sleep. A greater number of collisions were associated with reductions in total sleep time and REM sleep, whereas greater overall locomotor load correlated with increased REM sleep time and proportion. However, the intensity of locomotor load should be considered as high‐speed distance was linked to reduced REM sleep time. These findings underscore the need for ongoing sleep monitoring during the season, especially postmatch. Practitioners should tailor sleep strategies based on individual match data and contextual factors (e.g., match location) to ensure players have sufficient sleep opportunity.

### 4.1 Collisional Events Diminish Sleep Duration and REM Sleep

Players involved in more collisions during matches exhibited significant associations with reduced total sleep time and REM sleep (time and proportion), partially confirming our hypotheses. These observations align with existing literature linking contact load to alterations in postmatch sleep architecture; however, they differ in the specific sleep stages affected (Aloulou et al. 2020). Although a previous study reported that increased collision frequency was associated with reductions in deep sleep (and increases in light sleep) (Aloulou et al. 2020), we primarily observed changes in REM sleep.

Considering that REM sleep predominately occurs in the latter half of the sleep period and is interspersed with bouts of light sleep (Lee‐Chiong 2006) (which also decreased in our study; *β* = −0.9 min for every collision; *medium* effect and *p* = 0.296), the reduction in REM may be a result of reduced total sleep quantity. One potential explanation for reduced total sleep (and REM sleep) is the collisional activity performed during the match. Severe collisions, such as those resulting in a sport‐related concussion (mild traumatic brain injury), are known to induce neurological changes that alter sleep‐wake patterns. Even in the absence of a concussion diagnosis, collisional activity may still cause axonal damage, potentially affecting sleep (Donahue and Resch 2024). Interestingly, although studies assessing acute sleep responses to sport‐related concussions are limited (Stevens et al. 2022), existing evidence suggests that sleep quantity may increase to facilitate glymphatic drainage (Raikes and Schaefer 2016)—a finding contrary to our results. Nonetheless, our findings indicate that recurrent contact, even if subconcussive (i.e., indirect head impacts involving forceful head acceleration), may elicit changes in sleep architecture.

Notably, this study includes data from a player (a forward) who sustained a concussion diagnosis during a match. However, post hoc analysis (by removing the concussed player) reveals trends similar to those presented in our results, with both total sleep time (*β* = −4 ± 1 min and *p* = 0.009) and REM sleep (proportion: *β* = −0.6 ± 0.2% and *p* < 0.001; time: *β* = −2 ± 0 min and *p* = 0.016) decreasing for every match collision. Given the high prevalence of concussion incidents in professional rugby (Cosgrave and Williams 2019; Kemp et al. 2021), future research should consider employing home‐based PSG to assess the consequences of concussions on sleep architecture.

An alternate explanation for the observed reductions in total sleep time and REM sleep is that players' anthropometric characteristics, rather than the collisional events itself, may have influenced sleep outcomes. For instance, positional comparisons reveal that (i) forwards presented higher body mass indices (BMIs) than backs (30.7 ± 3.4 vs. 27.3 ± 1.5; *p <* 0.01), (ii) forwards experienced shorter sleep durations than backs (∼4.8 h vs. ∼5.9 h) and (iii) forwards executed more collisions in a match than backs (23 ± 12 vs. 10 ± 4; Table 2). Although evidence suggests that individuals with higher BMIs tend to experience shorter sleep durations, this has predominantly been observed in sedentary individuals (Vorona et al. 2005). In athletic cohorts, particularly in professional rugby, higher BMIs are most likely attributed to greater muscle mass rather than fat mass, which may elicit different sleep outcomes. This assumption warrants further investigation in future studies.

Given the critical role of sleep, particularly REM sleep, in restoring cognitive function and emotional regulation, the reductions observed in players with greater contact loads may suggest potential impairments in attention, tactical memory and motivation (Driller et al. 2023; D. J. Miller et al. 2022; Vyazovskiy and Delogu 2024). Scheduled activities the following morning (e.g., return travel and medical check‐ins) that require teams to wake earlier may further disrupt the latter half of sleep as was likely the case in one of our matches. Since forwards engage in more collisional activity than backs, they may benefit from additional sleep time. High‐performance staff might consider this when planning training and recovery to preserve postmatch sleep. Adjusting morning routines and implementing staggered medical check‐ins could allow backs (or lower‐contact load players) to commence first, extending sleep opportunities for players with greater contact loads (Swinbourne et al. 2016). Additionally, encouraging daytime naps may help mitigate the consequences of disrupted nocturnal sleep (Lastella et al. 2021). Such interventions could support cognitive and emotional restoration, ultimately enhancing player performance and wellbeing. Although these suggestions are informed by our findings, future research is needed in a greater number of matches to confirm these associations and to evaluate the effectiveness of such interventions in supporting player recovery postmatch.

### 4.2 Importance of Considering Velocity Thresholds for Locomotor Load on Sleep

Our results demonstrate mixed associations between total locomotor load and high‐speed locomotor load. Increased total distance was significantly associated with greater REM sleep time and proportion. In contrast, high‐speed running (> 5 m.s^−1^) was linked to reduced REM sleep time (−7 ± 3 min per 100 m), partially confirming our hypotheses. We also observed *large* effects, albeit not statistically significant, between greater high‐speed running and reduced REM sleep proportion (*β* = −2.7 ± 0.9% for every 100 m, *η*_*p*_^2^ = 0.72 and *p* > 0.05) as well as delayed REM onset latency (*β* = +12 ± 7 min for every 100 m, *η*_*p*_^2^ = 0.75 and *p* > 0.05).

The mechanisms linking locomotor load to REM sleep remain unclear. Nonetheless, several theories suggest that varying exercise intensities may elicit differing sleep responses, which have been shown in examining the effect of evening exercise on sleep in nonathletic populations (Stutz et al. 2019). One theory suggests that exercise, particularly at higher intensities, prioritises physical restoration achieved during deep sleep, thereby delaying REM sleep until later in the night (Shapiro et al. 1981; Frimpong et al. 2021). However, our data did not show significant associations with deep sleep. Another theory posits that high‐intensity exercise directly inhibits or delays REM sleep by increasing aminergic neurotransmitter levels and sympathetic activity (Lo Martire et al. 2020; Amatruda et al. 1975). This is supported by findings in elite athletes linking reduced REM sleep to elevated noradrenaline excretion rates (Netzer et al. 2001). Due to the field‐based nature of our study during competition, we could not directly assess these mechanisms leaving their role uncertain. Nonetheless, given that backs typically accumulate the largest high‐speed distances in professional rugby union (Sheehan et al. 2022), our findings potentially indicate their increased vulnerability to reduced REM sleep.

No significant associations were found between total sprint distance (> 7 m.s^−1^) and sleep outcomes. As shown in Table 2, players in our study accumulated relatively low sprint distances (0–229 m) and further analysis revealed seven match observations with no sprints exceeding > 7 m.s^−1^. This suggests that sprint run load may not be a major concern for sleep outcomes in professional rugby union. However, this relationship warrants further investigation in sports with greater sprint demands, such as Australian rules football (Varley et al. 2014), where athletes experience more sleep fragmentation and reduced total sleep during competition compared to rugby union players (D. J. Miller et al. 2017). Future studies in professional rugby should consider individualised velocity thresholds (relative to maximal velocity) (Reardon et al. 2015) or cardiometabolic thresholds to better understand the impact of high‐velocity locomotor load on postmatch sleep architecture. This approach could provide more nuanced insights into how sprint distances and other high‐intensity exercises influence sleep outcomes.

### 4.3 Consideration for an Individualised Recovery Approach for Players Within a Team

Given the substantial variability in match demands, it is important to recognise the differing sleep responses individuals may experience within a professional team. For instance, the total distance covered during a match ranged from 2019 to 8394 m (Table 2). Although positional differences (i.e., forwards vs. backs; Table 2) account for some of this variability, considerable differences also exist within positional groups, particularly between the starting lineup (*starters*) and substitute players (*finishers*). In our study, *finishers* had considerably less match exposure than *starters* (*finishers:* 24 ± 9 min and *starters:* 61 ± 16 min), which resulted in fewer collisions (10 ± 4 vs. 21 ± 13) and lower locomotor loads across total distance (3673 ± 948 m vs. 5801 ± 1553 m), high‐speed distance (254 ± 216 m vs. 458 ± 245 m) and sprint distance (25 ± 48 m vs. 62 ± 75 m). It may be that *finishers* are less susceptible to match‐induced reductions in REM sleep and total sleep time compared to *starters*. For teams without access to GPS and video analysis data, simply prioritising tailored sleep strategies for *starters* more than *finishers* may help the team recover from higher match demands.

### 4.4 Impact of Match Fixtures and Schedules

Although limited, our data suggest that match location significantly influenced sleep patterns but not sleep stages consistent with existing literature (Vitale et al. 2022; Sanders et al. 2024). In our study, the home match was associated with later bed and wake times (*p <* 0.05) compared to away matches, differing slightly from previous findings that expected earlier bedtimes (Fullagar et al. 2015). This discrepancy may be due to the home match having the latest kick‐off time (19:00) compared to away matches (15:00 and 15:35 local time). Unexpectedly, kick‐off times did not influence sleep, contrary to prior literature (Aloulou et al. 2020). This may indicate that we may lack sufficient volume or variation of data to draw firm conclusions on the effect of match schedules. Regardless, these findings highlight the need to contextualise each match, considering factors, such as match location (including return travel for away matches), as well as individual physical demands to ensure adequate sleep opportunities for recovery. Furthermore, sleep education and hygiene strategies can help players recover regardless of match location.

### 4.5 Limitations

Several limitations must be acknowledged. Firstly, contact load in this study was quantified solely on frequency counts, without accounting for collision intensity or placement (first point of contact, i.e., head, torso and leg). Although the advent of head acceleration assessment via mouthguard‐mounted inertial measurement units is in its infancy, this technology holds promise in quantifying contact load (frequency and intensity) in a more detailed manner. If budgets permit, future research should leverage these tools to better assess how collision intensity or placement may impact postmatch sleep, improving our understanding of sleep architecture and paving the way for an improved understanding of postmatch recovery. Secondly, as a field‐based study, we could not control for factors, such as caffeine or alcohol intake before sleep, and participants were not screened for sleep disorders, which are prevalent in rugby union players (Dunican et al. 2019)—all of which may have influenced sleep, particularly REM sleep. Future studies may include questionnaires for such insight. Thirdly, our analysis was restricted to three matches, limiting our ability to account for contextual factors, such as location and kick‐off times, alongside factors, such as jetlag and travel fatigue, all of which may have affected sleep. Future studies with larger samples and additional observations would help confirm our findings. A larger dataset may consider including nonmatch sleep data as well as analysing the time zone of matches as a variable or keeping the data exclusive to home matches only. Finally, although we chose to address multicollinearity by removing highly correlated variables to enhance interpretability and practical translation, we acknowledge that alternate data‐reduction techniques (e.g., partial least squares regression) may have identified different predictors.

### 4.6 Practical Applications

- A multidimensional approach that integrates both contact and locomotor load is essential for accurately quantifying the physical demands in professional rugby union matches. Utilising automated video analysis, real‐time GPS data and in‐season sleep monitoring can help with identifying players who may benefit from additional sleep strategies for postmatch recovery.
- Teams should carefully plan morning routines, particularly after a match. By adjusting the order of medical check‐ins, players with greater collisional activity and high‐intensity locomotor loads can start later, potentially enabling sleep extension and preserving sleep opportunities.
- Greater total locomotor load may increase REM sleep proportion and time. However, different velocity thresholds should be considered as the relationships with sleep architecture may differ with high‐speed and sprint distances.
- Practitioners should contextualise recovery decisions by considering individual physical match demands and match location as both factors can significantly alter sleep after a match.

## 5 Conclusion

Match demands, including both collision frequency and locomotor load, significantly influence postmatch sleep architecture in professional male rugby union players, particularly REM sleep. More collisional events are associated with reductions in total sleep time and REM sleep (proportion and time). Mixed associations with locomotor load on REM sleep outcomes emerge when considering velocity thresholds: total distance increased REM sleep (proportion and time), whereas high‐speed running (> 5 m.s^−1^) decreased REM sleep time. These findings underscore the importance of in‐season sleep monitoring and the use of readily available physical match data, alongside contextual factors (i.e., match location), to personalise sleep strategies for postmatch recovery.

## Conflicts of Interest

The authors declare no conflicts of interest.

## Supporting information


**Table S1:** Definition of sleep variables as well as the clinical significance threshold for total sleep time, sleep efficiency, sleep onset latency, wake after sleep onset and awakenings according to the American Academy of Sleep Medicine (AASM)^1^.
