# Supplementary material for: Heavy Hitters, Light Sleepers: Collision Frequency and Locomotor Load on Sleep Architecture in Professional Rugby Union Players
Source: Eur J Sport Sci. 2025 Aug 30;25(9):e70052. doi: 10.1002/ejsc.70052 (PMC12397986; doi:10.1002/ejsc.70052)
Supplement: Supplementary file 1 — Table S1: Definition of sleep variables as well as the clinical significance threshold for total sleep time, sleep efficiency, sleep onset latency, wake after sleep onset and awakenings according to the American Academy of Sleep Medicine (AASM)1. [file EJSC-25-e70052-s001.docx]

| **Table S1.** Definition of sleep variables as well as the clinical significance threshold for total sleep time, sleep efficiency, sleep onset latency, wake after sleep onset, and awakenings according to the American Academy of Sleep Medicine (AASM)^1^. | | |
| --- | --- | --- |
| **Sleep parameters** | **Unit of measure** | **Definition** |
| Total sleep time | min | The total duration of epochs scored as sleep (sum of N1, N2, N3 and REM). |
| Sleep efficiency | % | The percentage of time in bed spent sleeping. Total sleep time divided by total time in bed, multiplied by 100. |
| Sleep onset latency | min | The time from "lights off" to the first epoch of sleep. |
| Wake after sleep onset | min | The total time awake after the onset of sleep, until the final awakening. |
| Awakenings | n | Return to wakefulness after sleep onset; multiple epochs may be scored as wake. |
| Sleep onset time | hh:mm | The first epoch scored as any stage of sleep (N1, N2, N3, or REM). |
| Sleep offset time | hh:mm | The final epoch scored as any sleep stage (N1, N2, N3, or REM) before the final continuous period of wakefulness. |
| REM latency | min | The time from sleep onset to the first epoch of REM sleep. |
| Light sleep (time) | min | Total time spent in stage N1 and N2 from initial sleep onset, until final awakening. |
| Deep sleep (time) | min | Total time spent in stage N3 from initial sleep onset, until final awakening. |
| REM sleep (time) | min | Total time spent in rapid eye movement sleep from initial sleep onset, until final awakening. |
| Light sleep (proportion) | % | Proportion of time spent in N1 and N2 sleep, each scored according to AASM scoring rules^1^. |
| Deep sleep (proportion) | % | Proportion of time spent in N3 sleep, each scored according to AASM scoring rules^1^. |
| REM sleep (proportion) | % | Percentage of time spent in REM sleep, each scored according to AASM scoring rules^1^. |
| **Abbreviations:** non-rapid eye movement (NREM), rapid eye movement (REM), NREM stage 1 (N1), NREM stage 2 (N2), NREM stage 3 or slow-wave sleep (N3), American Academy of Sleep Medicine (AASM), electroencephalography (EEG), electromyography (EMG). | | |

**References**

1. Troester MM QS, Berry RB, et al. *The AASM Manual for the Scoring of Sleep and Associated Events: Rules, Terminology, and Technical Specifications (Version 3). American Academy of Sleep Medicine*. 2023.
